# Effects of cerium oxide nanoparticle on liver oxidative stress and morphological changes in opium withdrawal rats

**DOI:** 10.1016/j.toxrep.2025.102025

**Published:** 2025-04-09

**Authors:** Ebrahim Abbasi, Fatemeh Mirzaei, Ali Ghaleiha, Mona Pourjafar, Mehrdad Ahmadi, Seyed Somayeh Mirzajani

**Affiliations:** a Department of Clinical Biochemistry, School of Medicine, Hamadan University of Medical Sciences, Hamadan, Iran; b Department of Anatomical Sciences, School of Medicine, Hamadan University of Medical Sciences, Hamadan, Iran; c Behavioral Disorders and Substance Abuse Research Center, Institute of Neuroscience and Mental Health, Hamadan University of Medical Sciences, Hamadan, Iran; d Department of Molecular Medicine and Genetics, School of Medicine, Hamadan University of Medical Sciences, Hamadan, Iran

**Keywords:** Opium, Liver, Malondialdehyde, Addiction

## Abstract

Since oxidative stress increased during opioid withdrawal, this study aimed to evaluate the effects of cerium oxide nanoparticles (CeONP) on oxidative stress markers in rat livers. Male Wistar rats were randomly divided into 3 groups, including 1: control rats, 2: withdrawal group, and 3: withdrawal rats received CeONP. Opium administration was administered for 30 days. Then the withdrawal period started and CeONP (0.1 mg/kg) was administrated intravenously for 14 days. After that, the liver was removed, and serum was prepared. The expression of superoxide dismutase (SOD), glutathione peroxidase (GPx), and catalase (CAT) were measured by Real-time PCR. The amount of malondialdehyde (MDA), total oxidant status (TOS), total antioxidant capacity (TAC), and glutathione levels were determined by the available kit. Blood glucose levels and liver enzymes were measured by using the colorimetric method. A light microscope evaluated liver morphological changes. The liver biomarkers (ALT and AST) and glucose levels significantly increased in the withdrawal group (P < 0.05). Furthermore, the MDA and TOS increased dramatically in the withdrawal group (P < 0.001). However, the levels of GSH and TAC were reduced in the withdrawal group(P < 0.05). The gene expressions and activities of SOD, CAT, and GPx were reduced in the withdrawal group (P < 0.05). However, treatment of withdrawal rats with CeONP normalized all these factors(P < 0.05). CeONP restored histological alterations in the liver. Administration of opium causes an increase in oxidative stress and the activity of liver enzymes. However, treatment of withdrawal rats with CeONP showed potential hepatoprotective effects.

## Highlights

• The activity and expression of superoxide dismutase, glutathione peroxidase, and catalase increased in withdrawal rats.
• The MDA and TOS significantly were increased and GSH and TAC were reduced in the withdrawal group.
• CeONP normalized oxidative stress markers in withdrawal rats.
• CeONP restored histological alterations of the liver.

## Graphical Abstract

**Figure:**
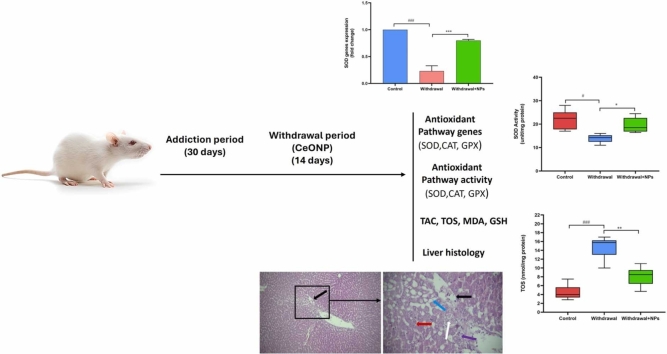

## 1 Introduction

The liver is the largest organ in the body and involves many physiological processes such as carbohydrate, lipid, and protein metabolism. This organ produces plasma proteins, bile acids, insulin, and storage vitamins. In addition, this organ plays a fundamental role in detoxifying and excreting a wide range of external and internal compounds. Since the liver is involved in the metabolism of many drugs, this tissue is directly exposed to damage [1]. Opium, like many other drugs, is metabolized in the liver and therefore can affect the function and structure of this tissue. Drug addiction is one of the most challenging health problems in many countries. Opium is the second most used substance in Asian countries. Hence, dependence on opium is one of the world's health problems, especially in the Middle East, and causes various individual, social, and economic problems [2].

Opium has been shown to induce inflammation [3] and oxidative stress in various body tissues, especially in the liver [4]. A study by Salahshoor et al., showed that morphine causes liver tissue damage and increases liver enzymes[5]. It has been showed that opium increases reactive oxygen species (ROS), such as hydrogen peroxide (H_2_O_2_), superoxide radicals, and hydroxyl free radicals[6]. Increased production of free radicals can initiate lipid peroxidation and ultimately produce lipid peroxidation products (MDA; malondialdehyde), which are involved in various disorders, including atherosclerosis, aging, diabetes, and liver damage[7].

Studies in human and animal models showed that opium withdrawal has some side effects. For example, it has been reported that morphine withdrawal in rats induces liver damage[8]. Continuous drug abuse induces adaptive mechanisms in the body and disrupts the function of neurons and neural networks. Tolerance, dependence, and sensitization are examples of adaptive mechanisms that develop with opiate use. These changes make addicts vulnerable for years after opium withdrawal [9]. Although the exact mechanism of opioid dependence and withdrawal syndrome is not yet well understood, it seems that the balance in the function of many neurotransmitters is disrupted during withdrawal [9]. It has been reported that the levels of sodium (Na), calcium (Ca), uric acid (UA), blood urea nitrogen (BUN), creatinine, and triglyceride increase, and thrombin time decreases in withdrawal cases compared to control [10]. It has been established that the levels of lipid peroxidation (MDA) increased and GSH, superoxide dismutase (SOD), and catalase (CAT) were decreased in the morphine withdrawal rats [8].

Recently cerium oxide nanoparticles (CeONP) have gained potential attention because of their interesting characteristics such as biofilm inhibition, redox activity, anti-inflammatory effects, antibacterial activity, etc. Cerium oxide (CeO_2_) in the bulk form exists in both + 3 and + 4 states, which helps them form CeO_2_ and CeO_2−*x*_ and consequently shows antioxidant effects. Hence, CeONP antioxidant effects are attracted by the electropositive charge surface. This nanoparticle efficiently binds to free radicals such as hydroxyl radicals, singlet oxygen, superoxide anion radicals, and hydrogen peroxide, and thus reduces oxidative stress. In this respect, oxidase, superoxide dismutase (SOD), glutathione peroxidase (GPx), and catalase (CAT) are only a few of the bio-relevant functions that this nanoparticle mimics to neutralize different ROS species[11]. Therefore, CeONP is accepted as an efficient agent against illnesses induced by oxidative stress due to its antioxidant properties [12]. Since oxidative stress increased during opioid withdrawal, this study aimed to evaluate the effects of CeONP on liver oxidative stress markers in animal rats.

## 2 Materials and methods

### 2.1 Animals

In this experimental study, 21 male Wistar rats weighing about 200–220 g were randomly divided into 3 groups (n = 6–7). The animals were kept in normal conditions, at a temperature of 20–24 °C and a light/dark cycle of 12:12 hours, and free access to water and food. The experimental groups included 1: control rats, 2: withdrawal group, and 3: withdrawal rats who received CeONP. Opium administration was administered for 30 days. Then the withdrawal period started and CeONP (0.1 mg/kg) was administrated intravenously for 14 days [13].

At the end of the study, the animals were anesthetized with ketamine (75 mg) and xylazine (10 mg/kg). The livers of the animals were removed, and a small portion was placed in 10 % formalin for histological analysis. The other portion was washed in normal saline and stored at −80°C freezer until more analysis. All the procedures for this experiment were approved by the Ethics Committee of Hamadan University of Medical Sciences (IR.UMSHA.REC.1396.762).

### 2.2 Addiction induction

To induce addiction, increasing concentrations of opium (10–40 mg/kg) were administrated orally to animals. Briefly, the rats received 10 mg of opium on day one, 15 mg on day two, 20 mg on day three, 25 mg on day four, 30 mg on day five, and 35 mg on day six. Then, 40 mg/kg remained constant for one month. Opium was dissolved in one milliliter of water and given to rats orally (gavage) at 8:00 AM and 2:00 PM [14]. To confirm addiction, naloxone (2 mg/kg) was injected intraperitoneally into one to two addicted rats in each group based on the previous experiment [15]. After injection animals show behavioral changes such as diarrhea, runny nose, head and wet-dog shaking, chewing, crawling, paw tremor, jumping, ptosis, and teeth chattering [15]. Animals that received naloxone were excluded from the study.

After one month of addiction, the withdrawal period started and CeONP (0.1 mg/kg) (NanoSany Co., Mashhad, Iran) was administrated intravenously for 14 days based on previously published papers [13], [16], [17]. CeONP dispersed in a normal saline solution.

### 2.3 Biochemical factors

The blood was centrifuged at 2000 rpm for 5 minutes and the serum separated. The activity of liver enzymes, including alanine aminotransferase (ALT) and aspartate aminotransferase (AST), and glucose levels were determined by colorimetric method using available kits (Pars Azmoon, Iran).

### 2.4 Lipid peroxidation

MDA was measured using the thiobarbituric acid (TBA) method according to previous articles [18]. Briefly, liver tissue was first homogenized, then the supernatant was separated after centrifugation (20,000 rpm for 15 minutes). To 50 μl of the resulting homogenate, 4 ml of distilled water and 1 ml of TBA reagent were added, and the reaction mixture was heated in a boiling water bath for 45 minutes. After cooling, 5 ml of n-butanol was added to the above mixture, and the tubes were shaken well. The samples were centrifuged (20,000 rpm for 20 minutes), the butanol-colored phase was separated, and the fluorescence intensity was measured by fluorimetry at an excitation wavelength of 515 nm and an emission wavelength of 553 nm [18].

### 2.5 The amount of total oxidant (TAC)

2.5. TAC levels were measured by ferric ferric-reducing antioxidant power (FRAP) assay. In this method, ferrous iron is oxidized to ferric iron in a medium with moderate acidity, which reacts with xylanol orange[19]. Briefly, 50 μL of the sample was mixed with 95 μL of reagent (25 mM sulfuric acid, 150 μM xylyl orange, and 100 mM sorbitol) and vortexed. Then, the sample was incubated for 30 minutes at room temperature and centrifuged. Then, the absorbance of the sample was read at a wavelength of 560 nm.

### 2.6 Reduced glutathione (GSH)

The GSH concentration was measured using Ellman’s reagent (5,5′-dithiobis-[2-nitrobenzoic acid] or DTNB) as an indicator. In brief, the homogenized sample was added to Ellman’s reagent, and its absorbance was measured at 412 nm. GSH level was measured by using the available kit (ZellBio GmbH, Germany).

### 2.7 The amount of total antioxidant (TAC)

TAC measurement was performed by the FRAP method based on the previous paper [18]. In this method, antioxidant compounds at low pH cause the reduction of the ferric tripyridyltriazine complex (Fe III TPTZ) to the Fe II form. Briefly, 100 μl of the sample was added to 3 ml of FRAP reagent and then read at a wavelength of 593 nm.

### 2.8 Activities of antioxidant enzymes

Toxic superoxide radicals are generated during oxidative processes that can damage various tissues. The SOD enzyme converts toxic superoxide radicals (O_2_^◦^) into H_2_O_2_ and hydrogen. This method uses xanthine and xanthine oxidase to produce superoxide radicals, which react with 2-iodophenyl 3-nitrophenol 5-phenyltetrazolium (I.N.T) to produce a red formazan color. SOD activity is measured by measuring the degree of inhibition of this reaction [20]. SOD activity was measured by using the available kit (ZellBio GmbH, Germany).

The catalase enzyme decomposes H_2_O_2_. By decomposition of H_2_O_2_ in the UV region, the absorption at a wavelength of 240 nm decreases. The amount of catalase activity can be determined from the difference in absorption at different times. Catalase activity was measured using the available kit (ZellBio GmbH, Germany).

Glutathione peroxidase is one of the important antioxidant enzymes in the body that removes free radicals. In this reaction, H_2_O_2_ is decomposed in the presence of glutathione peroxidase. The measurement of enzyme activity is based on the oxidation of NADPH to NADP^+^, which is measured at a wavelength of 340 nm. Glutathione peroxidase was performed using the available kit (ZellBio GmbH, Germany).

### 2.9 Real-time PCR assay

Total RNA was extracted from the tissue by using Trizol (Invitrogen, Carlsbad, CA) according to the manufacturer's instructions. The purity and concentration of total RNA were determined by a Nano-Drop spectrophotometer. RNA integrity was evaluated by agarose gel electrophoresis. cDNA was synthesized by using a cDNA synthesis kit (Fermentas Life Sciences, Vilnius, Lithuania). The total RNA was extracted by Trizol reagent (Invitrogen, Carlsbad, CA). The purity and concentration of total RNA were determined by a Nano-Drop spectrophotometer (Thermo Fisher Scientific, USA). RNA quality was evaluated by agarose gel electrophoresis. Then, cDNA was synthesized using a cDNA synthesis kit (Fermentas, Vilnius, Lithuania) using 500 ng of total RNA. Then, synthesized cDNA was used for PCR reactions. Briefly, 1 µl of cDNA, 10 µl of SYBR green (QuantiTect SYBR® Green PCR Kits, Qiagen), 0.5 µl of forward primer, 0.5 µl of reverse primer, and 8 µl of nuclease-free water were used by Roche light cycler 96 (Roche Diagnostics, Germany). Glyceraldehyde 3 phosphate dehydrogenase (GAPDH) was used as a housekeeping gene. The threshold cycles (Ct) were determined and normalized with housekeeping gene (GAPDH). The fold changes (relative gene expressions normalized with GAPDH) were calculated using the Livak formula (2^-ΔΔCt^) as follows: ΔCt = Ct of target gene – Ct of housekeeping gene, ΔΔCt = test gene ΔCt – control ΔCt, and gene expression (fold change) = 2^−(ΔΔCt)^. Primers were used based on our previous experiment [21].

### 2.10 Histological procedures

For liver morphological examination, a small portion of the liver was fixed with 10 % formalin for 24 h. Samples were dehydrated and embedded in paraffin. After that, the 5-μm thickness was prepared and stained with hematoxylin-eosin (H & E) and evaluated by light microscopy.

### 2.11 Statistical analysis

All results are presented as Mean ± SEM. Comparisons between the groups were performed by one-way analysis of variance (ANOVA) followed by the Tukey test using the SPSS package (version 16, SPSS, Inc). The P values less than 0.05 were regarded as statically signiﬁcant. GraphPad Prism software (version 8) was used to draw graphs (Cullman or box plot graphs).

## 3 Results

### 3.1 Biochemical factors

**Fig. 1:**
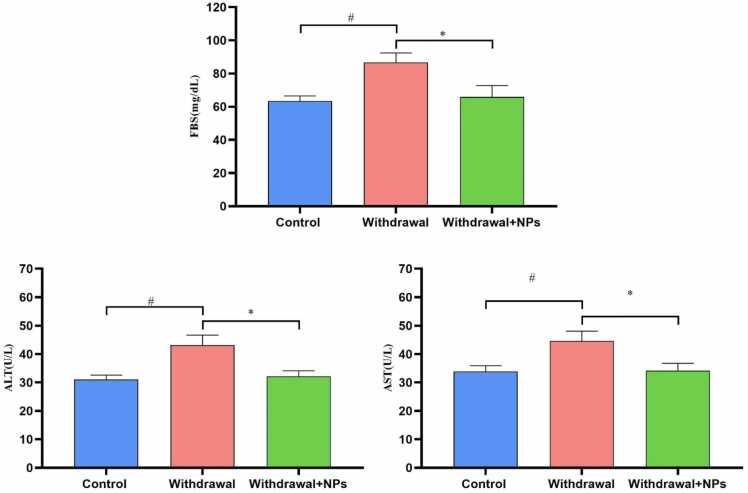
The blood glucose and liver enzymes in different groups. ^#^: p < 0.05 compared to the control group. * : p < 0.05 compared to withdrawal rats. The graphs show that the blood glucose and liver enzyme levels increased in withdrawal rats, while treatment with CeONPs reduced glucose levels and liver enzymes. ALT: alanine aminotransferase, AST: aspartate aminotransferase, FBS: fasting blood sugar.

The activity of liver enzymes, including ALT and AST were significantly increased in opium withdrawal rats when compared to normal rats (p < 0.05 and p < 0.05, respectively). Treatment of animals with CeONP leads to the reduction of ALT and AST as compared to control rats (p < 0.05 and p < 0.05, respectively). Moreover, the blood glucose levels were increased in opium withdrawal rats when compared to the control group (p < 0.05), while CeONP led to the reduction of blood glucose levels when compared to control rats (p < 0.05) (Fig. 1).

### 3.2 Oxidative stress markers

**Fig. 2:**
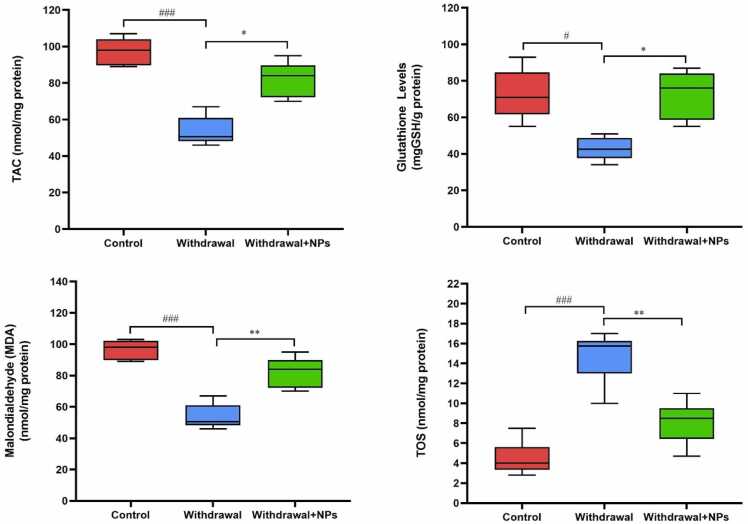
Oxidative stress markers in different groups. ^#^: p < 0.05, and ^###^: p < 0.001 compared to the control group. * : p < 0.05, and ^**^: p < 0.01 compared to withdrawal rats. Boxplots show that the amount of TAC and GSH were reduced, and TOS and MDA were decreased in withdrawal rats, while treatment with CeONPs normalized these markers. TAC: total antioxidant capacity and TOS: total oxidant status, MDA: malondialdehyde, GSH: glutathione.

There was a significant reduction in TAC and GSH levels in opium withdrawal rats when compared to normal rats (p < 0.001and p < 0.05, respectively). Treatment of opium withdrawal with CeONP caused a significant reduction in TAC and GSH levels when compared to untreated rats (p < 0.05 and p < 0.05, respectively) (Fig. 2).

In comparison with the control group, the level of MDA and TOS significantly reduced in withdrawal animals when compared with the control group (p < 0.001 and p < 0.001, respectively). Treatment of withdrawal animals with CeONP significantly reduced MDA and TOS levels in the liver as compared to control rats (p < 0.051 and p < 0.01, respectively) (Fig. 2).

### 3.3 Antioxidant enzymes activities

**Fig. 3:**
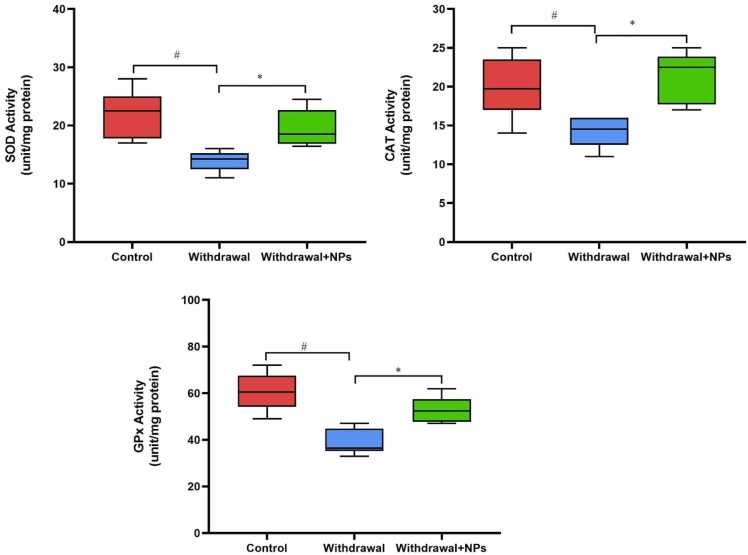
Activity of antioxidant enzymes in different groups. ^#^: p < 0.05 compared to the control group. * : p < 0.05 compared to withdrawal rats. Boxplots show that the activities of SOD, CAT, and GPx were reduced in withdrawal rats, while treatment with CeONPs increased the activities of these enzymes. SOD: superoxide dismutase, GPx: glutathione peroxidase, CAT: catalase.

The level of SOD activity in the liver tissue of withdrawal rats was significantly lower than in the control group (P < 0.05). The level of this enzyme activity significantly increased in the CeONP-treated group as compared with the untreated group (P < 0.05). The CAT activity was significantly reduced in opium withdrawal rats as compared with the control group (p < 0.05). However, administration of CeONP significantly increased CAT activity (p < 0.05). The level of GPX activity in the liver tissue was significantly reduced in opium withdrawal rats when compared control group (P < 0.05). Our results revealed that the treatment of withdrawal rats with CeONP significantly increased the level of GPX activity ‎‎(P < 0.05) (Fig. 3). ‎

### 3.4 Antioxidant genes expressions

**Fig. 4:**
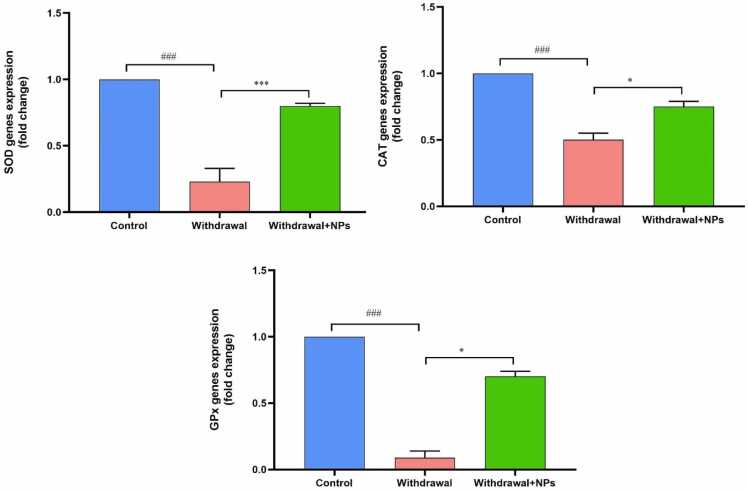
Expression of antioxidant enzymes in different groups. ^###^: p < 0.001 compared to the control group. ^***^: p < 0.001 compared to withdrawal rats. Graphs show that the expression of SOD, CAT, and GPx was reduced in withdrawal rats, while treatment with CeONPs increased the activities of these enzymes. SOD: superoxide dismutase, GPx: glutathione peroxidase, CAT: catalase.

The gene expression of SOD was significantly reduced in withdrawal rats (P < 0.001), while treatment with CeONP increased SOD gene expression (P < 0.001). Catalase (CAT) gene expression was reduced in opium withdrawal rats compared with the control group(P < 0.001). However, NPs treatment showed a marked rise in catalase gene expression as compared to withdrawal rats (P < 0.05). The gene expression of GPx also had a similar trend. Its levels were significantly reduced in withdrawal rats (P < 0.001), whereas its gene expression increased in NPs treated animals compared with control withdrawal rats (P < 0.05) (Fig. 4).

### 3.5 Histological changes

**Fig. 5:**
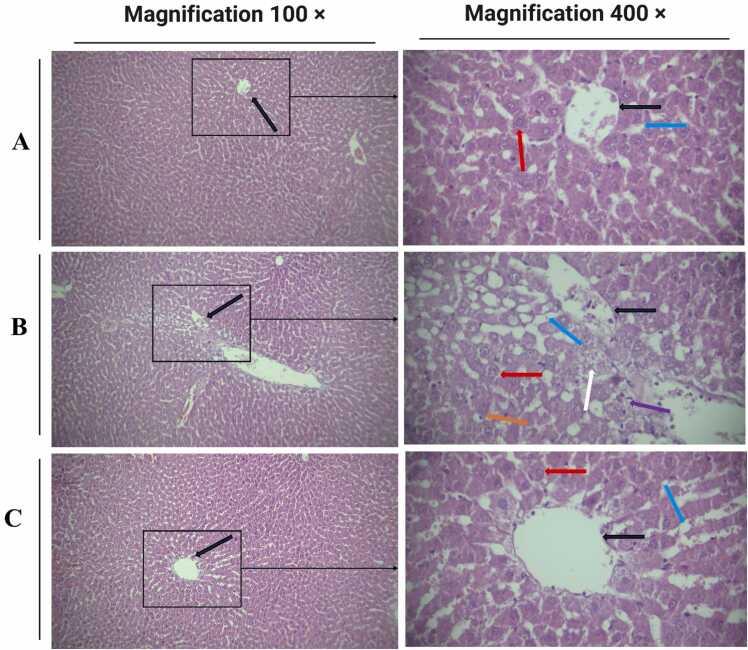
Liver morphological changes in different groups based on H&E staining. The liver sections of healthy rats showed normal structure, while destructive alterations were observed in withdrawal rats. Treatment with CeONP restored the morphological changes in the liver (magnification 100 × and 400 ×). Black arrow: central vein, blue arrow: sinusoidal space, red arrow: hepatocyte, purple arrow: mononuclear infiltration, orange arrow: kupffer cells, white arrow: foam cell. A: Control, B: Withdrawal, C: Withdrawal + CeONP.

The liver sections of healthy rats showed normal structure. The destructive alterations were observed in withdrawal rats. In the withdrawal group, the normal centric hepatocyte organization was altered, and the sinusoid liver was congested. Furthermore, mild mononuclear infiltration, congestion, and hyperplasia of Kupffer cells were observed in the opium withdrawal group compared with control. However, treatment with CeONP restored these morphological changes (Fig. 5).

## 4 Discussion

Previous studies have shown that opium withdrawal reduces antioxidant activity and increases free radical production [22]. In this study, the oxidative stress markers were increased in withdrawal rats. The levels of TAC and glutathione were significantly reduced in withdrawal rats, while MDA and TOS concentrations were increased. Treatment of withdrawal tars with CeONPs normalized all of these markers. The accumulation of free radicals causes damage to hepatocytes through impaired enzyme activity, lipid peroxidation, and DNA damage [23].

In this experiment, we reported that the activity of CAT, SOD, and CAT activities was significantly reduced, demonstrating a disruption of the liver antioxidant capacity that can lead to oxidative stress and damage to the liver in opium withdrawal rats. Our results showed that CeNPs restored both non-enzymatic components (GSH) and enzymatic antioxidant systems. Our results support previous studies reported that reduced antioxidant capacity. The result of Zhang et al., shows that morphine increases oxidative stress markers, such as MDA, and 8-OHdG, and reduces the glutathione peroxidase, SOD, and catalase in the rat liver [24]. Karajibani et al., [6] showed that ROS increased and the levels of TAC, GSH, vitamins C, E, and A, as well as the activities of CAT, SOD, and GPx were increased in opium addiction. Furthermore, it has been reported that opium increased ROS levels and reduced the activity of GPx, SOD, and CAT [25]. SOD is a first-line antioxidant enzyme that detoxifies O•_2_ into H_2_O_2_, which is converted into water by GPx and CAT. These enzymes protect cells from free radical attack. Previous experiment has demonstrated that opium reduces GSH concentration in hepatic mitochondria, induces the activation SOD, and reduces GPx and CAT activities [23]. However, treatment of rats with CeONP prevents opium-induced liver damage.

Since CeONPs mimic the SOD, GPx, and catalase, it can efficiently remove reactive oxygen species (ROS). The antioxidant properties of CeONPs are attributed to their intrinsic characteristics, made of divalent oxygen anions (O2 −), and cerium atoms in + 3 and + 4 oxidation states. In the catalytic redox reaction, Ce3 on the surface of cerium oxide nanoparticles can capture oxygen atoms from H2O2 and convert them to H2O, whereas Ce3 is oxidized to Ce4. During reducing situations, Ce4 can be reduced back to Ce3 by scavenging hydrogen peroxide radicals, or the intracellular redox of CeONPs can be maintained by choosing appropriate reducing agents (e.g., metallic reducing) [11]. Hence, CeONPs by electron transfer ability can eliminate free radicals and effectively protect cells from oxidative stress damage. Furthermore, the oxygen vacancies in CeNPs’ surface provide extra sites for redox activity and act as platforms for electron capture and transfer, further increasing their antioxidant properties [11], [26]. Ghazavi et al., showed that the serum levels of C3 and C4 complement factors, high-sensitivity C-reactive protein (hs-CRP) increased in opium-addicted people. However, the results show that addicted people had higher levels of TAC compared to control [27].

In this study, the glucose levels also increased in withdrawal rats. Activation of the hypothalamus-pituitary recognizes morphine withdrawal–adrenocortical (HPA) axis that increases stress hormones such as cortisol and corticosterone levels. These hormones are involved in anxiety and stress of withdrawal period and glucose levels. Increased these hormones are related to hyperglycemia. Based on previous experiments, blood glucose levels were altered in dependent animals [28].

Our results show that glucose levels increased in withdrawal rats. Park et al., reported that glucose levels were increased in morphine-dependent animals [29]. According to a previous report, blood glucose increases during the withdrawal period due to glucocorticoid secretion and stress conditions [28]. Hence, one mechanism of hyperglycemia during morphine or opium withdrawal syndrome is that stress-induced hypercortisolism may affect the pancreas, motivating glucagon release and thus leading to hepatic glucose production [28]. Opioids can increase blood glucose through various pathways, affecting glucoregulatory hormones such as glucagon, insulin, cortisol, and epinephrine. It has been reported that the secretion of insulin is decreased upon opioid injection [30].

It has been shown that oxidative stress (as seen in withdrawal rats) or increased levels of saturated fatty acids in the liver can cause phosphorylation of various target molecules, including the insulin receptor and insulin signaling, by activating the serine-threonine kinase cascade, and as a result, cause impaired insulin function and consequently lead to insulin resistance [31]. Therefore, there is a possibility of insulin resistance in addicted individuals in the long term. Radahmadi et al. [32] showed that morphine at high doses increases glucose production in the liver and reduces its clearance by peripheral tissues, thus causing hyperglycemia. Moreover, Ipp et al. [33] showed that morphine may cause hyperglycemia by increasing glucagon secretion. Gozashti et al. [34] showed that fasting glucose levels in addicted individuals increased significantly compared to the non-addicted group.

In this study, opium withdrawal led to significant increases in AST, ALT, and ALP levels compared to healthy rats. Bedair et al., reported that the activity of liver enzymes increased in morphine withdrawal rats [35]. However, administration of CeONPs normalized liver enzymes. Thus, the potential of CeONPs to normalize liver enzymes was probably attributed to their antioxidant activity.

In this study, morphological alteration was observed in the liver of opium withdrawal rats, while CeONPs normalized these changes. Manfared et al.,[36] by examining the effects of opium addiction on the tissue structure of rabbit liver, showed that opium caused central lobular vein hyperemia, leukocyte infiltration, fatty changes, hepatocyte necrosis, and dilation of hepatic sinusoids (47). As mentioned, CeONPs with potential antioxidant activity and inflammatory effects can restore liver injury. Shojaeepour et al., showed that opium used had higher lead levels compared to the control group. Lead poisoning can induce various behavioral, biochemical, and physiological dysfunctions in the body’s organs [37]. It has been reported that lead by increasing free radical and inflammatory markers production can lead to liver damage [38].

This study shows the hepatoprotective effects of CeONPs in opium withdrawal rats. However, there are some limitations. First, in this study, we did not use a positive antioxidant control group (e.g., N-acetylcysteine). By using this group, we could directly compare the efficacy of CeONPs with verified antioxidants. Second, although in this experiment, we aimed to evaluate the protective effects of CeONPs in opium withdrawal, we did not use opium alone group.

## 5 Conclusion

This study showed increased MDA and TOS levels and reduced TAC and glutathione levels in opium withdrawal rats. The activity and gene expression of CAT, SOD, and GPx were reduced in opium withdrawal rats. However, the effectiveness of CeONPs was demonstrated by normalizing the oxidative stress, blood glucose, and liver morphological changes.

## Funding

This study was funded by the 10.13039/501100004697Hamadan University of Medical Sciences (No: 9611177326).

## CRediT authorship contribution statement

**Mirzajani Seyed Somayeh:** Validation, Methodology, Investigation, Conceptualization. **Ahmadi Mehrdad:** Writing – original draft, Validation, Supervision, Methodology, Investigation, Data curation. **Pourjafar Mona:** Visualization, Validation, Methodology, Conceptualization. **Ghaleiha Ali:** Writing – review & editing, Visualization, Validation, Supervision, Investigation. **Mirzaei Fatemeh:** Writing – review & editing, Writing – original draft, Methodology, Data curation, Conceptualization. **Abbasi Ebrahim:** Writing – review & editing, Writing – original draft, Resources, Project administration, Methodology, Investigation, Funding acquisition, Formal analysis, Data curation.

## Declaration of Competing Interest

The authors declare the following financial interests/personal relationships which may be considered as potential competing interests: Ebrahim Abbasi reports financial support was provided by Hamadan University of Medical Sciences, Hamadan, Iran. EBRAHIM ABBASI reports a relationship with Hamadan University of Medical Sciences Faculty of Health and Research Center for Health Sciences Department of Environmental Health Engineering that includes: funding grants. EBRAHIM ABBASI has patent pending to NONE. If there are other authors, they declare that they have no known competing financial interests or personal relationships that could have appeared to influence the work reported in this paper.

## Data Availability

Data will be made available on request.
